# Cerebellar function in children with and without dyslexia during single word processing

**DOI:** 10.1002/hbm.24792

**Published:** 2019-10-09

**Authors:** Sikoya M. Ashburn, D. Lynn Flowers, Eileen M. Napoliello, Guinevere F. Eden

**Affiliations:** ^1^ Center for the Study of Learning, Department of Pediatrics Georgetown University Medical Center Washington District of Columbia USA

**Keywords:** cerebellum, child, dyslexia, functional magnetic resonance imaging, reading, reading disability, word processing

## Abstract

The cerebellar deficit hypothesis of dyslexia posits that dysfunction of the cerebellum is the underlying cause for reading difficulties observed in this common learning disability. The present study used functional magnetic resonance imaging (fMRI) and a single word processing task to test for differences in activity and connectivity in children with (*n* = 23) and without (*n* = 23) dyslexia. We found cerebellar activity in the control group when word processing was compared to fixation, but not when it was compared to the active baseline task designed to reveal activity specific to reading. In the group with dyslexia there was no cerebellar activity for either contrasts and there were no differences when they were compared to children without dyslexia. Turning to functional connectivity (FC) in the controls, background FC (i.e., not specific to reading) was predominately found between the cerebellum and the occipitaltemporal cortex. In the group with dyslexia, there was background FC between the cerebellum and several cortical regions. When comparing the two groups, they differed in background FC in connections between the seed region right crus I and three left‐hemisphere perisylvian target regions. However, there was no task‐specific FC for word processing in either group and no between‐group differences. Together the results do not support the theory that the cerebellum is affected functionally during reading in children with dyslexia.

## 1 INTRODUCTION

The learning disability developmental dyslexia impacts ~10% of the population and is characterized as a difficulty in learning to read accurately and fluently (Lyon, Shaywitz, & Shaywitz, 2003). Its cause is mainly attributed to difficulties mapping sounds of words (phonemes) to graphemes for the decoding of words (Bradley & Bryant, 1983; Bruck, 1992; Wagner & Torgesen, 1987), thereby also slowing the process whereby words are eventually recognized by sight. As such, the most prominent theory explaining dyslexia centers around a phonological processing deficit (Démonet, Taylor, & Chaix, 2004; Gabrieli, 2009; Peterson & Pennington, 2015; Stanovich, 2016; Vellutino, Fletcher, Snowling, & Scanlon, 2004) and this theory has received support from several different types of studies (McCardle, Scarborough, & Catts, 2001). First, there is research demonstrating that phonemic awareness predicts later reading outcome in children and that it is impaired in dyslexia (Bradley & Bryant, 1983; Peterson & Pennington, 2015; Wagner & Torgesen, 1987). Second, reading intervention approaches targeting phonological awareness have successfully brought about gains in reading for children with dyslexia (Alexander, Andersen, Heilman, Voeller, & Torgesen, 1991). Third, neuroimaging studies have converged on differences in brain activity in left lateralized cortical regions, mainly inferior frontal, temporal–parietal, and occipital‐temporal cortices, with the former two regions considered to be involved in phonological processing aspects of reading (Linkersdörfer, Lonnemann, Lindberg, Hasselhorn, & Fiebach, 2012; Maisog, Einbinder, Flowers, Turkeltaub, & Eden, 2008; Richlan, Kronbichler, & Wimmer, 2011).

The cerebellar deficit hypothesis of dyslexia posits that congenital cerebellar dysfunction is the cause of poor reading skills in this reading disability. Specifically, it has been proposed that abnormal cerebellar function impacts articulatory and phonological awareness skills, thereby causing reading difficulties (Nicolson, Fawcett, & Dean, 2001). The cerebellar hypothesis also holds that disruption of the cerebellum not only explains deficits in reading, but also in writing and spelling, as well as skills outside of language that are associated with the cerebellum and reported to be affected in dyslexia. These other skills include balance (C. J. Stoodley, Fawcett, Nicolson, & Stein, 2005), automaticity (R. Nicolson & Fawcett, 1990), habituation to eye‐blink conditioning (R. I. Nicolson, Daum, Schugens, Fawcett, & Schulz, 2002), and timing skills (Overy, Nicolson, Fawcett, & Clarke, 2003). However, a causal role of the cerebellum in dyslexia has not been substantiated (Zeffiro & Eden, 2001) and there has been criticism (Rack, Snowling, Hulme, & Gibbs, 2007) of treatments for dyslexia targeting cerebellar functions (e.g., balance exercises; Reynolds, Nicolson, & Hambly, 2003). Not only is the effectiveness of this approach controversial, but there are concerns that this displaces the learning of phonological skills considered by many to be critical to successful intervention (McCardle et al., 2001).

The role of the cerebellum in reading is not fully understood. An early review of neuroimaging studies on reading suggested activation of bilateral cerebellum in typical adults (Fiez & Petersen, 1998). Consistent with this, meta‐analyses of studies of reading in adults found convergence of activity in right cerebellum (Turkeltaub, Eden, Jones, & Zeffiro, 2002) and more recently in bilateral cerebellum (Martin, Schurz, Kronbichler, & Richlan, 2015) in addition to the left hemisphere perisylvian regions associated with reading. The report by Martin et al. (2015) also included a meta‐analysis of studies in children. However, there was no convergence of activation within the cerebellum in children. Thus, the cerebellum appears to be involved in reading in adults, but its function remains unclear in children. Importantly, very few neuroimaging studies have found functional differences in the cerebellum when comparing groups with and without dyslexia during reading and reading‐related tasks. Again, the most consistent findings from these studies are reflected in meta‐analyses. Two meta‐analyses of activation studies in dyslexia in children/adolescents did not report differences in the cerebellum (Maisog et al., 2008; Richlan et al., 2011). These meta‐analyses did report less activity in dyslexia in inferior frontal, temporal–parietal, and occipital‐temporal cortices, consistent with the widely‐accepted view of the role of dorsal and ventral cortical brain areas involved in reading (Pugh et al., 2000, 2001). A meta‐analysis by Linkersdörfer et al. (2012) that combined children and adults revealed greater, rather than less, activation in left lobule VI in dyslexia during reading‐related tasks. However, when meta‐analyses were conducted separately for children and adults on a subset of studies showing less activity in dyslexia, there were no differences between those with and without dyslexia in the cerebellum.

Altogether, there is limited evidence of a functional role of the cerebellum in typically reading children or aberrations in dyslexia. However, few have conducted a direct study of the cerebellar deficit hypothesis. Only one functional brain imaging study was explicitly conducted to test the cerebellar deficit hypothesis in dyslexia for English readers (R. I. Nicolson et al., 1999). For this positron emission tomography (PET) study the investigators focused on motor learning rather than reading, and found that adults with dyslexia had less activation than controls in right cerebellum during motor sequence tasks, both when performing a pre‐learned sequence and while learning a new sequence (R. I. Nicolson et al., 1999). However, it is important to examine and contrast the cerebellum in groups with and without dyslexia during reading.

Further, given the proposed mechanism of the cerebellar deficit hypothesis, which implies abnormal cerebro‐cerebellar loops, it is important to combine measures of cerebellar activity with measures of functional connectivity between the cerebellum and cortical regions known to be involved in reading. The cerebellum has a topographic organization of higher functions where motor, visual spatial, language, and working memory tasks are mapped onto discrete cerebellar sub‐regions in the posterior lateral cerebellar hemispheres (C. J. Stoodley, Valera, & Schmahmann, 2012; C. Stoodley & Schmahmann, 2009). Specifically, lobule VI and crus II are activated during language tasks (C. Stoodley & Schmahmann, 2009). As noted by Stoodley and colleagues (C. J. Stoodley, 2014), these specific regions have been shown to have functional connectivity with ventral attention and frontal–parietal networks as demonstrated in a resting‐state functional magnetic resonance imaging (fMRI) study (Buckner, Krienen, Castellanos, Diaz, & Yeo, 2011), including cortical areas involved in reading, namely occipital‐temporal cortex, inferior frontal gyrus, superior temporal gyrus, angular gyrus, and supramarginal gyrus. In the same vein, Alvarez and Fiez (2018) used a reverse inference approach to show that right lobule VIIb/crus II has intrinsic functional connectivity with cortical regions involved in phonology, namely the intraparietal cortex. Connections between cerebellum and cortex are contralateral; thus, language‐related cerebellar regions are predominately right lateralized (Mariën et al., 2013). Taken together, the cerebellum is organized topographically for a variety of tasks, including language, and this organization is reflected in its functional connections to the cortex. Booth, Bebko, Burman, and Bitan (2007) found functional connections between right cerebellum and left inferior frontal and left lateral temporal cortex in typical adults during a rhyme judgment task. In a neuroanatomical study employing diffusion tensor imaging, Travis, Leitner, Feldman, and Ben‐Shachar (2015) found fractional anisotropy of the cerebellar peduncles in children and adolescents to be correlated with reading skills. Most relevant to the current study, in Chinese children with dyslexia, Feng et al. (2017) found differences in activity and functional connectivity in the cerebellum. However, to date there have been no studies comparing functional connectivity of the cerebellum during reading in an alphabetic language in children with and without dyslexia, although such an approach would be important to test the cerebellar deficit hypothesis of dyslexia.

Using fMRI we tested whether the cerebellum is active during single word processing in typical children and those with dyslexia, and conducted between‐group comparisons. For these analyses, we used (a) a whole cerebellum followed by (b) a priori cerebellar sub‐region of interest approach. We examined the contrast of single real word (RW) processing versus fixation, as well as single real word versus false font (FF) processing. The latter contrast identifies activity related specifically to word processing rather than other aspects of the task (e.g., button pressing). For functional connectivity (FC), we examined background functional connectivity to identify intrinsic connections of the cerebellum. This was followed by generalized psychophysiological interactions (gPPI) regression analysis, to determine cerebellar FC specific to single word processing. For both FC analyses, we examined each group and computed between‐group differences. Overall, we expected typical readers to show cerebellar activation during reading (in lobule VI, crus I, crus II, and/or lobule VIIb) and to have functional connectivity between the cerebellum and left inferior frontal gyrus specific to single word processing. Should the cerebellar deficit hypothesis hold true, we would expect differences in activity and functional connectivity during word processing in children with dyslexia in comparison to typical readers.

## 2 METHODS AND MATERIALS

### 2.1 Participants

Children with dyslexia were recruited from a school that specializes in teaching children with reading disability. They and the typical readers were monolingual, native English speakers. All except for two participants were right‐handed, one left‐hander in each group. Children with ADHD were included in the study, however a student *t*‐test comparing between‐group Connors' index T‐score found no significant difference of ADHD symptoms between the two groups. Subsets of these participants were included in prior publications on dyslexia (Evans, Flowers, Napoliello, & Eden, 2014; Evans, Flowers, Napoliello, Olulade, & Eden, 2014; Krafnick, Flowers, Luetje, Napoliello, & Eden, 2014; Krafnick, Flowers, Napoliello, & Eden, 2011; O. A. Olulade, Flowers, Napoliello, & Eden, 2015; Olumide A. Olulade, Flowers, Napoliello, & Eden, 2013). Participants were given informed consent prior to beginning the study and all protocols were approved by the Georgetown University Institutional Review Board.

All children completed a battery of behavioral testing including the Wechsler abbreviated scale of intelligence (WASI; Wechsler, 1999) for measures of IQ. From the Woodcock‐Johnson III Tests of Achievement (WJ‐III; Woodcock, McGrew, & Mather, 2001) the Word Identification and Word Attack subtests were used to assess single real‐ and pseudo‐ word reading ability, respectively. Reading rate was assessed using the Reading Fluency subtest (Woodcock et al., 2001). Digit Span from the Wechsler Intelligence Scale for Children (WISC IV; Wechsler, 2003) was used to assess working memory and rapid automatized naming (RAN; Denckla & Rudel, 1976a, 1976b) was used to assess fluency of naming letters and numbers. All participants had a standard score of 80 or above on the Full‐scale WASI IQ test. Children in the control group were required to have a standard score above 92 on both real‐ and pseudo‐ word reading subtests of the WJ‐III. Children with dyslexia were required to have a standard score of 92 or below on either the real‐ or pseudo‐word reading test on the WJ‐III and these children had a documented history of their reading disability, with most attending a school that specializes in the teaching of children with learning disabilities. Typical readers were recruited as age‐matched controls. After addressing head movement during the scans (described below), several children were excluded from the original sample of 39 children with dyslexia and 28 without dyslexia, leaving 23 in the group with dyslexia (9 females, 14 males, mean age = 9.8, *SD* = 1.4) and 23 children in the control group (13 females, 10 males, mean age = 9.7 years, *SD* = 1.8). A summary of the mean group demographic and behavioral data for participants in the study can be found in Table 1. The groups did not differ in age. As expected, children with dyslexia had significantly lower reading scores as well as lower naming fluency and working memory scores. Additionally, the two groups differed in Verbal and Performance IQ. Therefore, Performance IQ was used as a covariate of no interest in the between‐group analyses of the fMRI data.

**Table 1:** Demographics and behavioral assessments

	Controls	Dyslexics	*p*‐value
N	23	23	–
Sex (F/M)	13/10	9/14	–
Age (years)	9.7 ± 1.8	9.8 ± 1.4	
WASI Verbal IQ^a^	120.6 ± 14.5	110.3 ± 10.2	.008
WASI Performance IQ^a^	114.0 ± 13.1	100.0 ± 10.8	.002
WJ‐III Word ID^b^	115.5 ± 12.4	77.5 ± 9.6	<.001
WJ‐III Word Attack^b^	110.4 ± 12.6	89.6 ± 11.2	<.001
WJ‐III Reading Fluency^b^	119.5 ± 20.3	73.1 ± 14.1	<.001
RAN (Letters)	111.1 ± 15.2	87.2 ± 9.0	<.001
Digit Span	104.7 ± 16.2	92.0 ± 12.7	.020

*Note*: Scores reported as averages of standard scores ±*SD*. *p*‐values are listed for student *t*‐test for between‐group differences. Significance determined if *p* < .05.

a Denotes tests from the Wechsler abbreviated scale of intelligence (WASI).

b Denotes tests from the Woodcock Johnson‐III (WJ‐III).

### 2.2 fMRI task and procedure

Participants performed an implicit reading task (Price, Wise, & Frackowiak, 1996), which consisted of visually presented RW and FF conditions. Participants were asked to press a button in their right hand if a tall feature was present (e.g., eaten or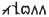) and a button in their left hand if no such feature was present (e.g., manor or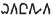) as accurately and quickly as possible. RW stimuli were single five‐letter, low frequency words (O. A. Olulade et al., 2015; Turkeltaub et al., 2004; Turkeltaub, Gareau, Flowers, Zeffiro, & Eden, 2003). FF stimuli provided an active control condition and were created by manipulating the letters from the RW stimuli to create new, unfamiliar characters (Arial font letters were cut and the sections reconnected to generate the FF). As such, the number of elements and angles were similar across the Real Word and False Font conditions. Also, the FF stimuli were matched to the RW stimuli for both length and location of ascenders and descenders. RW and FF stimuli were presented in separate blocks, always alternating with a block of fixation. During fixation blocks (Fix), children were instructed to keep their eyes on the cross‐hair in the center of the screen. We examined RW > Fix as a way to gauge general activation to the task and RW > FF to identify activity specific to single word processing.

Each participant completed two runs and each run consisted of two blocks of each task condition (RW and FF), with 10 stimuli per block. Both runs were used in the final analysis for all participants except for two control subjects, where of the two runs, one was removed due to excessive motion (see below). The presentation of each stimulus was 1.2 s and was followed by a fixation cross that was presented for 3 s. Each task block had a duration of 42 s while interleaving fixation blocks had a duration of 18 s blocks. Therefore, the overall length of the run was 4 min and 27 s. The number of brain volumes acquired was the same for the RW, FF, and Fix conditions (28 volumes each). We used presentation software (Neurobehavioral Systems Inc., Albany, CA) for stimulus presentation and recording responses. We collected reaction time (RT) and accuracy for both conditions. RT and accuracy were compared between the groups using a two‐sample student *t*‐test (Table 2). One control participant did not have in‐scanner performance data due to a technical malfunction.

**Table 2:** Participant in‐scanner performance

	Controls	Dyslexics	*p*‐value
Accuracy (% correct)			
Total accuracy	90.1% ± 7.4	89.5% ± 7.6	.439
Real words	90.1% ± 8.6	91.4% ± 6.8	.556
False fonts	90.2% ± 7.2	87.5% ± 10.8	.316
RW/FF difference	0.1% ± 5.8	4.0% ± 9.6	.104
Response time (ms)			
Total reaction time	925.9 ± 130.5	1,011.4 ± 157.4	.053
Real words	924.4 ± 139.8	1,022.3 ± 178.7	.047
False fonts	927.3 ± 126.6	1,000.2 ± 142.1	.076
RW/FF difference	0.3 ± 49.3	22.0 ± 73.4	.249

*Note:p*‐values are listed for student *t*‐test for between‐group differences. Significance determined if *p* < .05.

Abbreviations: FF, false font; RW, real word.

Prior to the scanning session, all participants practiced the task in a mock scanner to become habituated to both the task and scanning environment. At the conclusion of the actual scanning session, a pencil‐and‐paper test was performed in which participants were asked whether they had seen a given stimulus during the scans (as in Turkeltaub et al., 2003). There were 40 targets and 40 foils, for each condition.

### 2.3 Image acquisition

All functional scans were acquired on a 3 T Siemens Trio scanner, located at the Center for Functional and Molecular Imaging at Georgetown University. Functional images were obtained with a T2*‐weighted echo planar imaging sequence using Flip Angle = 90°, TR = 3 s, TE = 30 ms, and 50 axial slices (2.8 mm with a 0.2 mm gap), FOV = 192 mm, in‐plane resolution = 64 × 64, resulting in 3 mm cubic voxels. All functional images covered the whole brain, including complete coverage of the cerebellum.

### 2.4 Data analysis

There were three methods used to analyze the data. First, we examined activity in the cerebellum during single word processing in comparison to Fixation (RW > Fix) and also in comparison to the active control task, False Fonts (RW > FF). Next, we performed two types of functional connectivity analyses: background FC (Norman‐Haignere, McCarthy, Chun, & Turk‐Browne, 2012) and generalized psychophysiological interactions (gPPI) FC. The former provides insight to how the cerebellum may be intrinsically connected to cortical regions independent of the task. The latter distinguishes whether these functional connections are specific to single word processing. For all three analyses, we generated within‐group and between‐group maps. We constrained the analyses to the cerebellum, as described in detail next.

#### 2.4.1 Preprocessing

For all analyses (fMRI activity and FC) preprocessing steps were completed with Statistical Parametric Mapping, version 12 (SPM12; Welcome Department of Cognitive Neurology, London). The toolboxes SUIT (Diedrichsen, Balsters, Flavell, Cussans, & Ramnani, 2009) and Voxel Based Morphometry segmentation (Ashburner & Friston, 2000) were also used for activation and functional connectivity analyses, respectively. All data were individually inspected for gross artifacts and to ensure full cerebellum coverage. The first five functional images of each run were discarded. Functional images were slice‐time corrected, realigned, and coregistered to the anatomical data.

All data were corrected for head movement using ArtRepair (ART; https://www.nitrc.org/; adjusted in‐house). Time points with scan‐to‐scan motion greater than 0.75 mm (25% of the voxel size) were regressed out during statistical analysis. The percentage of scans regressed out in this way did not differ between the two groups, *p* > .05. An entire run was removed if more than 25% of the scans exceeded either the 0.75mm motion threshold or a 1.5% global signal change threshold.

#### 2.4.2 Functional activation in the cerebellum and sub‐regions of the cerebellum

After preprocessing, we ran first‐level GLM analysis on the functional data, thereby generating contrast images for each subject (RW > Fix and RW > FF). We then used SUIT to isolate the cerebellum. This step involved the generation of a cerebellar mask for each participant, which was quality controlled and overlaid onto the T1‐anatomical image within MRICron (Rorden, Karnath, & Bonilha, 2007). Manual corrections were performed as needed. Careful attention was given to the border between the cerebellum and cerebrum to avoid including voxels in adjacent inferior occipital or temporal cortex. We then normalized the anatomical image into SUIT space. The resulting deformation field was then used to transform the fMRI data into SUIT space by re‐slicing the statistical maps of the activation data. Lastly, these normalized images were smoothed with a 4 × 4 × 4‐mm full‐width height maximum Gaussian kernel.

To test particular sub‐regions within the cerebellum, we created masks for left and right lobule VI, crus I, crus II, and lobule VIIb (total of eight regions). These sub‐regions were chosen based on the literature (Linkersdörfer et al., 2012; Moore, D'Mello, McGrath, & Stoodley, 2017; C. J. Stoodley et al., 2012; C. Stoodley & Schmahmann, 2009) and defined within the SUIT atlas (Diedrichsen et al., 2009). We used small volume correction (SVC) at the second‐level to conduct the region of interest (ROI) analyses for each sub‐region. We also used a Bonferroni‐correction to account for the use of multiple ROIs, such that the adjusted threshold for significance was p‐FWE‐Bonferroni < .00625. Throughout this article, we use the term “cerebellar sub‐region(s)” to refer to these ROIs used in the functional activation analyses.

#### 2.4.3 Functional connectivity analyses

After preprocessing (described above) the data were segmented using Voxel Based Morphometry segmentation (Ashburner & Friston, 2000), and normalized to MNI space. We then used the CONN toolbox 16.b (Whitfield‐Gabrieli & Nieto‐Castanon, 2012) for both the background and task‐specific functional connectivity analyses. For each, we performed denoising with simultaneous regression of temporal confounding factors, as well as temporal filtering on the unsmoothed functional data. The temporal confounding factors included six head position parameters, a vector to indicate whether a particular scan was preceded by our 0.75 mm threshold (whereby scans preceded by inter‐scan head motion <0.75 mm received a 0 and scans preceded by inter‐scan head motion greater than or equal to 0.75 mm received a 1), and block conditions (RW, FF, and Fixation), convolved with canonical hemodynamic response function. The CONN toolbox also estimated principal components from subject‐specific white matter and CSF masks, which were created during the VBM segmentation step detailed above. Both white matter and CSF had five principal components per subject.

First, we performed a *background functional connectivity* (Norman‐Haignere et al., 2012) correlation analysis, as previously used in brain imaging studies (Fair et al., 2007), to provide insight into the cerebellum's intrinsic FC with the cortex. This approach regresses out the effects of task blocks over the run to generate a measure of intrinsic brain connectivity. Thus, we regressed the effects of RW, FF, and Fixation. Next, we applied a low band‐pass filter (.008 to .09 Hz). First‐level analysis was performed using a GLM, HRF weighting, and bivariate correlation parameters for the ROI‐to‐ROI analysis. ROIs were chosen based on the literature (described in more detail below) and while we use the term ROI here, the sequential sections refer to these as “cerebellar seed regions” and “cortical target regions.” First‐level analyses were run for a left and right set of each cerebellar seed region (lobule VI, crus I, crus II, and lobule VIIb) with all the cortical target regions (i.e., right and left lobule VI was run with all the cortical target regions). Second‐level analysis was performed on each cerebellar seed. For example, right lobule VI seed was tested with its homolog and seven cortical target regions. Note that we did not otherwise test for functional connectivity within cerebellar seeds (i.e., cerebellum to cerebellum).

Second, we performed *gPPI regression analyses*, which provides insight into task‐specific connectivity, that is, FC modulated by single word processing. We applied a high band‐pass filter (.008 to Inf Hz). The first‐level ROI‐to‐ROI analyses were performed using gPPI and bivariate regression parameters for a left and right set of each cerebellar seed region (lobule VI, crus I, crus II, lobule VIIb). The gPPI regression analysis builds each task condition into the regression model, that is, RW and FF. Second‐level analyses were performed on each cerebellar seed for the contrast of RW > FF. For example, right lobule VI seed was tested with its homolog and seven cortical target regions. Again, we did not test for functional connectivity between cerebellar seeds (i.e., cerebellum to cerebellum).

**Figure 1:**
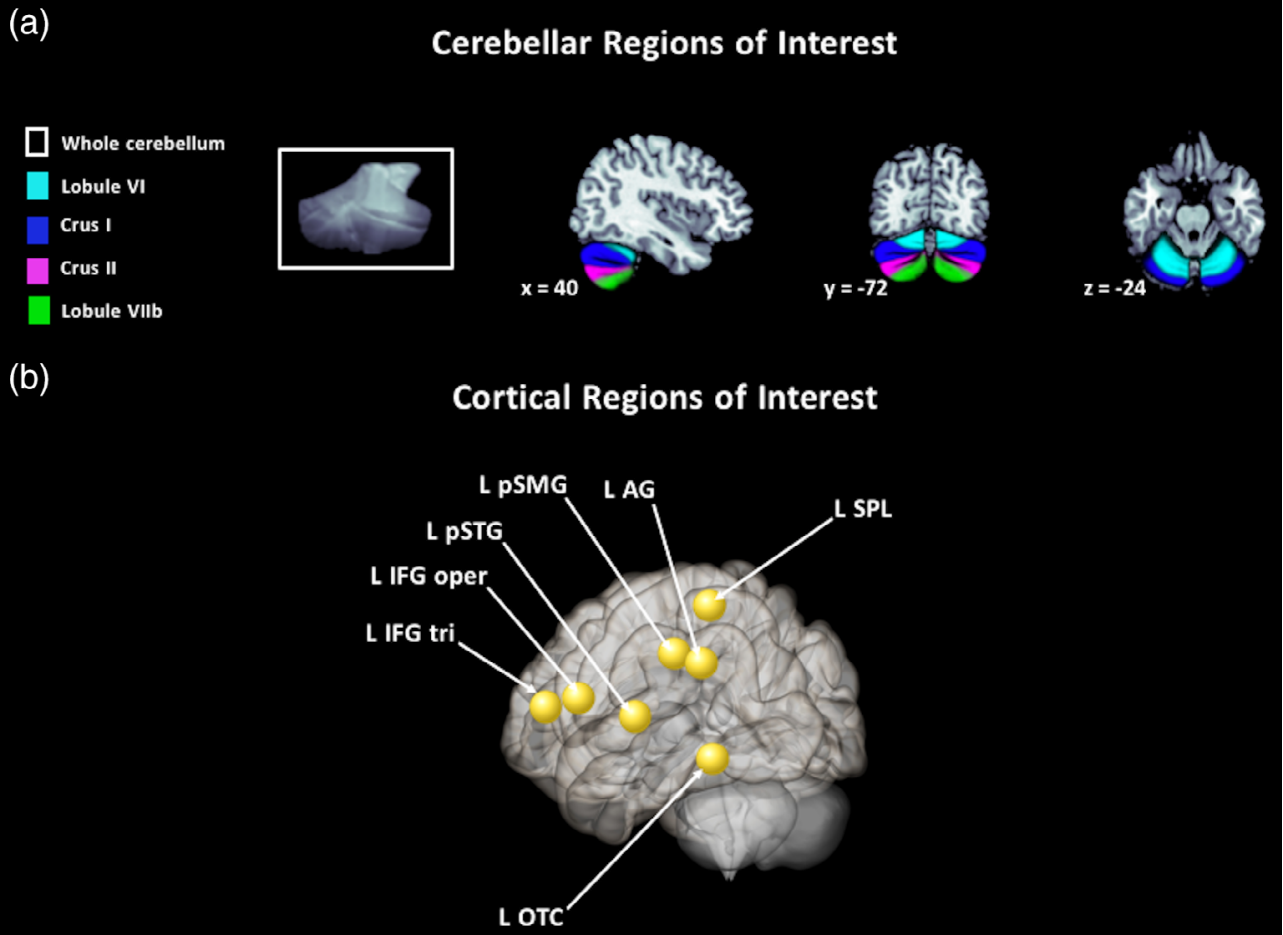
Cerebellar and cortical regions of interest used for the activation and connectivity analyses. (a) Cerebellar regions chosen based on the literature and defined with the SUIT atlas (Diedrichsen et al., 2009). These were used in the activation analysis (cerebellar sub‐regions) and again in the connectivity analyses (cerebellar seed regions). (b) Cortical target regions for the functional connectivity analyses are shown as spheres but all were anatomical regions derived from the FSL Harvard‐Oxford Atlas (Desikan et al., 2006)

*Cerebellar seed regions* for the connectivity analyses in both background and gPPI analyses were the same eight cerebellar sub‐regions as described above for the activation analyses, chosen based on the literature: bilateral lobule VI, crus I, crus II, and lobule VIIb (Figure 1). Cortical *target regions* were chosen based on the traditional reading network as defined by Pugh et al. (2001) and the meta‐analysis by Martin et al. (2015). Specifically, we selected the following eight left hemisphere regions within CONN (Harvard‐Oxford atlas; Desikan et al., 2006): inferior frontal gyrus *pars triangularis* (IFG tri), inferior frontal gyrus *pars opercularis* (IFG oper), posterior superior temporal gyrus (pSTG), superior parietal lobule (SPL), posterior supramarginal gyrus (pSMG), angular gyrus (AG), and occipital‐temporal cortex (OTC). These regions can be found in Figure 1.

Both within‐ and between‐group significance was determined with p‐FDR = .05, seed‐level correction, two‐sided statistic. All connectivity results were visualized with CONN toolbox and overlaid with spheres to optimize the visibility of the seed and target regions.

## 3 RESULTS

### 3.1 Behavioral measures

Accuracy and reaction times for both groups are shown in Table 2. Of most interest are the between‐group comparisons of the subtractions between the RW and FF conditions, as these are the conditions contrasted for the activation analysis to identify areas specific to word processing (RW > FF). There were no between‐group differences in these values for accuracy or reaction times, as shown in previous studies (O. A. Olulade et al., 2015; Turkeltaub et al., 2003). The post‐scan pencil‐and‐paper test used to assess the participants' familiarity with stimuli found that both groups performed significantly above chance when identifying RW but not FF stimuli, indicating participants had processed the word stimuli during the scan.

### 3.2 Functional activation constrained to (a) the whole cerebellum and (b) cerebellar sub‐regions

#### 3.2.1 Controls

**Figure 2:**
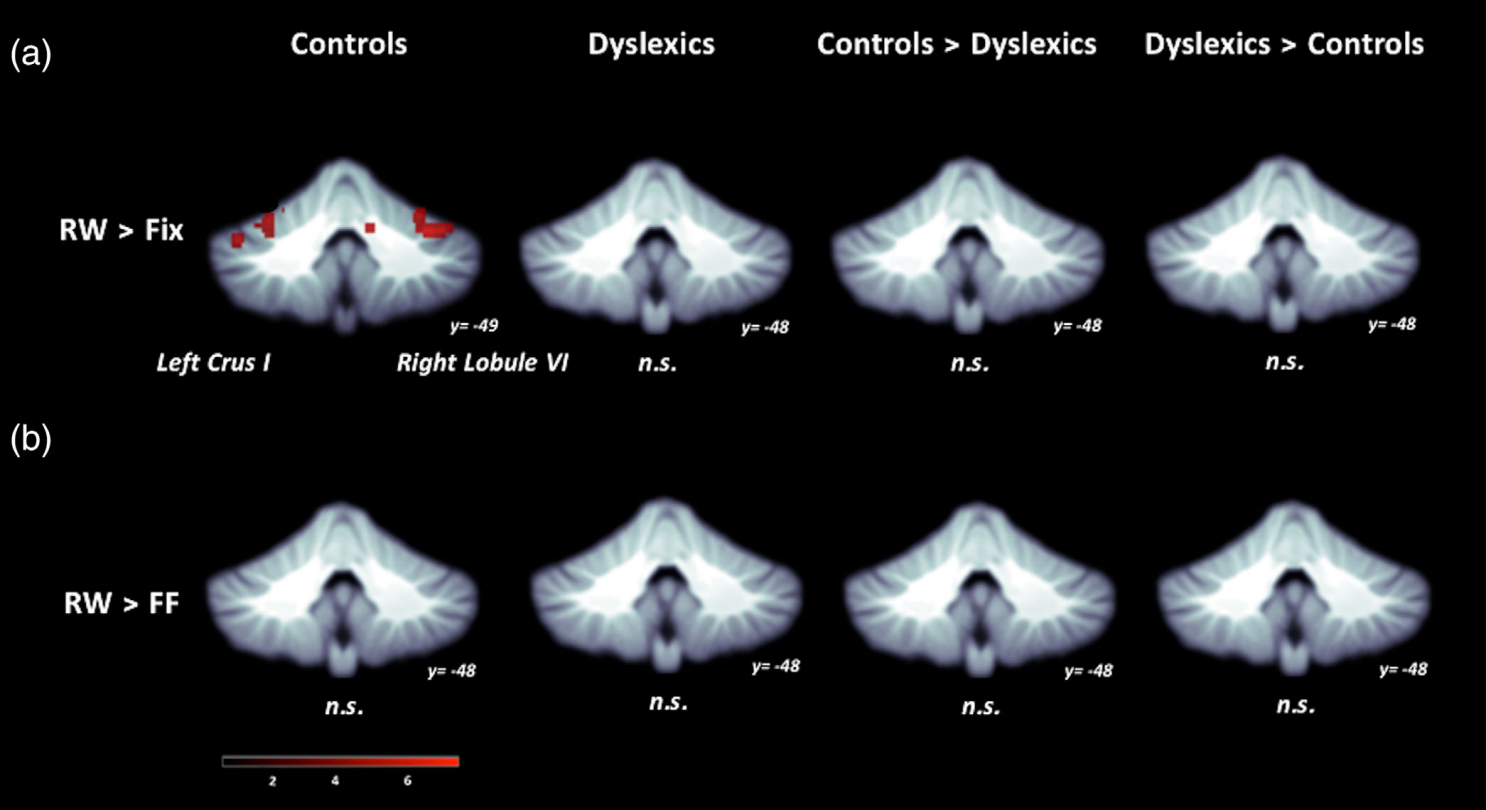
Cerebellar functional activation maps. (a) Real Word > Fixation and (b) Real Word > False Font contrasts. Significant activation in vermis VI (not shown), left crus I, and right lobule VI in Control group, height threshold *p* < .001, *p* < .05 FWE‐corrected. No activation for Real Word > False Font for either group and no between‐group differences for either contrast (A or B)

**Figure 3:**
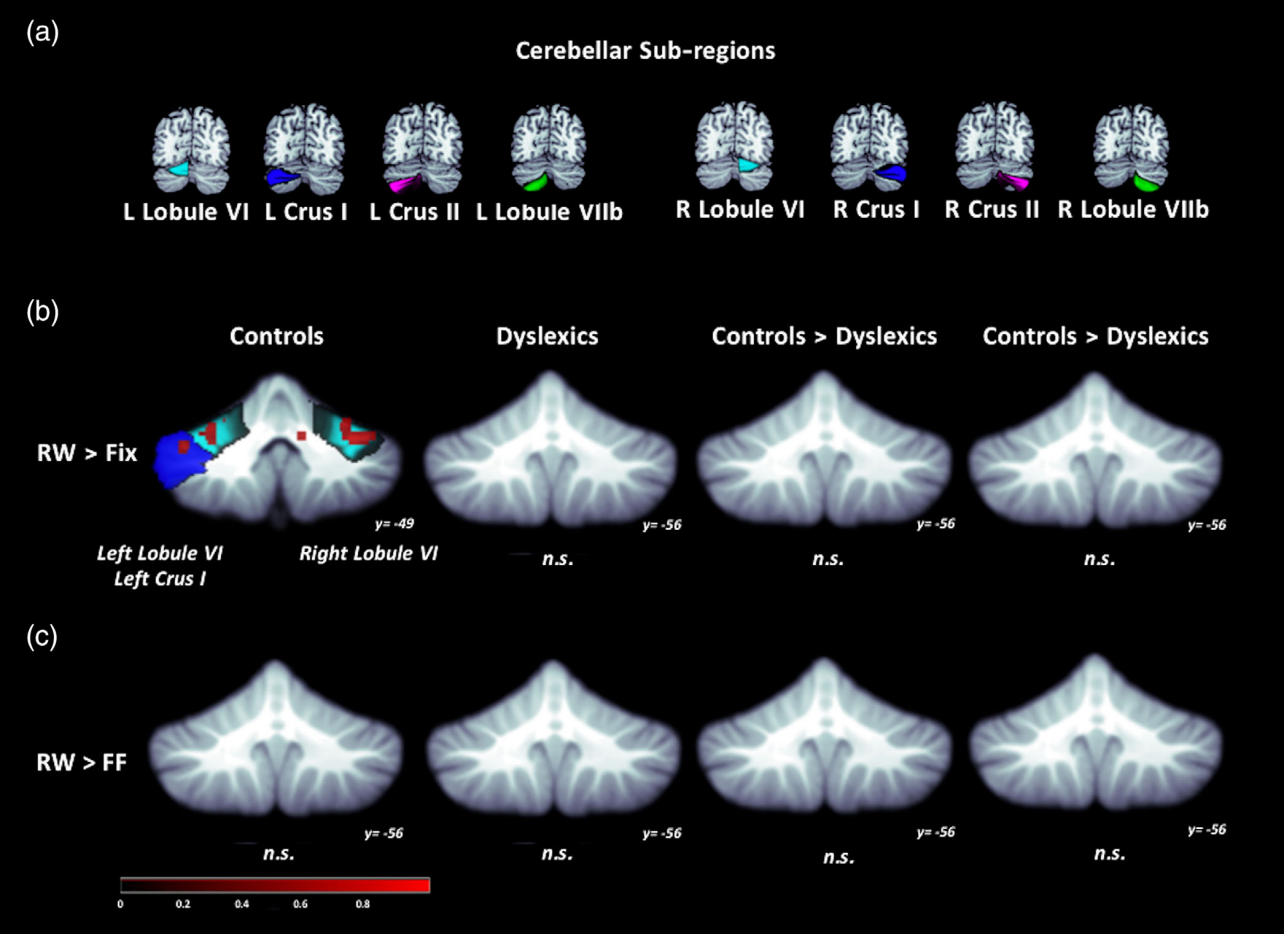
Functional activation maps constrained to eight cerebellar sub‐regions. (a) Location of the cerebellar sub‐regions: bilateral lobule VI, crus I, crus II, and lobule VIIb. (b) Real Word > Fixation and (c) Real Word > False Font contrasts. Significant activation in left lobule VI, left crus I, and right lobule VI for Real Word > Fixation in controls. Height threshold *p* < .001, *p*‐FWE < .05 and Bonferroni‐corrected so that significance was *p* < .00625. No significant activation for Real Word > False Font in Controls, Dyslexics, nor between‐group differences. Corresponding coordinates in Table 4

For the analysis conducted at the level of the whole cerebellum, the group of typical readers had activation for the RW > Fixation contrast in vermis VI, left crus I, and right lobule VI (Figure 2; Table 3). However, for the contrast of RW > FF (designed to find activity specific for reading) there was no significant activation. For the analysis examining RW > Fixation in the eight cerebellar sub‐regions, we found activation in left lobule VI, left crus I, and right lobule VI in the control group (Figure 3a; Table 4). Critically, for RW > FF, the controls had no significant activation in any cerebellar sub‐regions.

**Table 3:** Functional activation results for the whole cerebellum analysis

		MNI coordinates			Volume		
Group	Contrast	*x*	*y*	*z*	(voxels)	*p*‐value	Anatomical region
Controls							
	*RW > Fix*	−2	−76	−16	500	<.001	Vermis VI
		−50	−56	−32	268	<.001	Left crus I
		32	−52	−28	200	<.001	Right lobule VI
	*RW > FF*		*None*				
Dyslexics							
	*RW > Fix*		*None*				
	*RW > FF*		*None*				
Controls > dyslexics							
	*RW > Fix*		*None*				
	*RW > FF*		*None*				
Dyslexics > controls							
	*RW > Fix*		*None*				
	*RW > FF*		*None*				

*Note*: Significance determined by height threshold = .001, *p* < .05 FWE‐corrected. “*None*” indicates no significant findings.

Abbreviations: FF, false font; RW, real word.

**Table 4:** Functional activation results for the cerebellar sub‐region analyses

			MNI coordinates			Volume	
Group	Cerebellar sub‐regions	Contrast	*x*	*y*	*z*	(voxels)	*p*‐value
Controls							
	*Left lobule VI*	*RW > Fix*	−32	−38	−26	143	<.001
			−2	−76	−18	120	<.001
		*RW > FF*					*n.s*.
	*Right lobule VI*	*RW > Fix*	32	−52	−28	189	<.001
		*RW > FF*					n.s.
	*Left crus I*	*RW > Fix*	−50	−56	−32	151	<.001
		*RW > FF*					*n.s*.
	*Right crus I*	*RW > Fix*					*n.s*.
		*RW > FF*					*n.s*.
	*Left crus II*	*RW > Fix*					*n.s*.
		*RW > FF*					*n.s*.
	*Right crus II*	*RW > Fix*					*n.s*.
		*RW > FF*					*n.s*.
	*Left lobule VIIb*	*RW > Fix*					*n.s*.
		*RW > FF*					*n.s*.
	*Right lobule VIIb*	*RW > Fix*					*n.s*.
		*RW > FF*					*n.s*.
Dyslexics							
	*All sub‐regions*	*RW > Fix*					*n.s*.
		*RW > FF*					*n.s*.
Controls > dyslexics							
	*All sub‐regions*	*RW > Fix*					*n.s*.
		*RW > FF*					*n.s*.
Dyslexics > controls							
	*All sub‐regions*	*RW > Fix*					*n.s*.
		*RW > FF*					*n.s*.

*Note*: Significance determined by height‐threshold <.001, *p*‐FWE < .05 and Bonferroni‐corrected for multiple cerebellar sub‐regions. “*n.s*.” indicates no significant findings.

Abbreviations: FF, false font; RW, real word.

#### 3.2.2 Dyslexics

The within‐group maps for the group with dyslexia showed no significant activation when contrasting RW > Fixation or RW > FF at the level of the whole cerebellum, or at the level of cerebellar sub‐regions.

#### 3.2.3 Controls versus dyslexics

Comparison of whole cerebellar functional activation between the two groups (controls greater than dyslexics and vice versa) revealed no difference in cerebellar activation for RW > Fixation or RW > FF at the level of the whole cerebellum, or at the level of cerebellar sub‐regions.

### 3.3 Background functional connectivity analysis of cerebellar seed regions

#### 3.3.1 Controls

**Figure 4:**
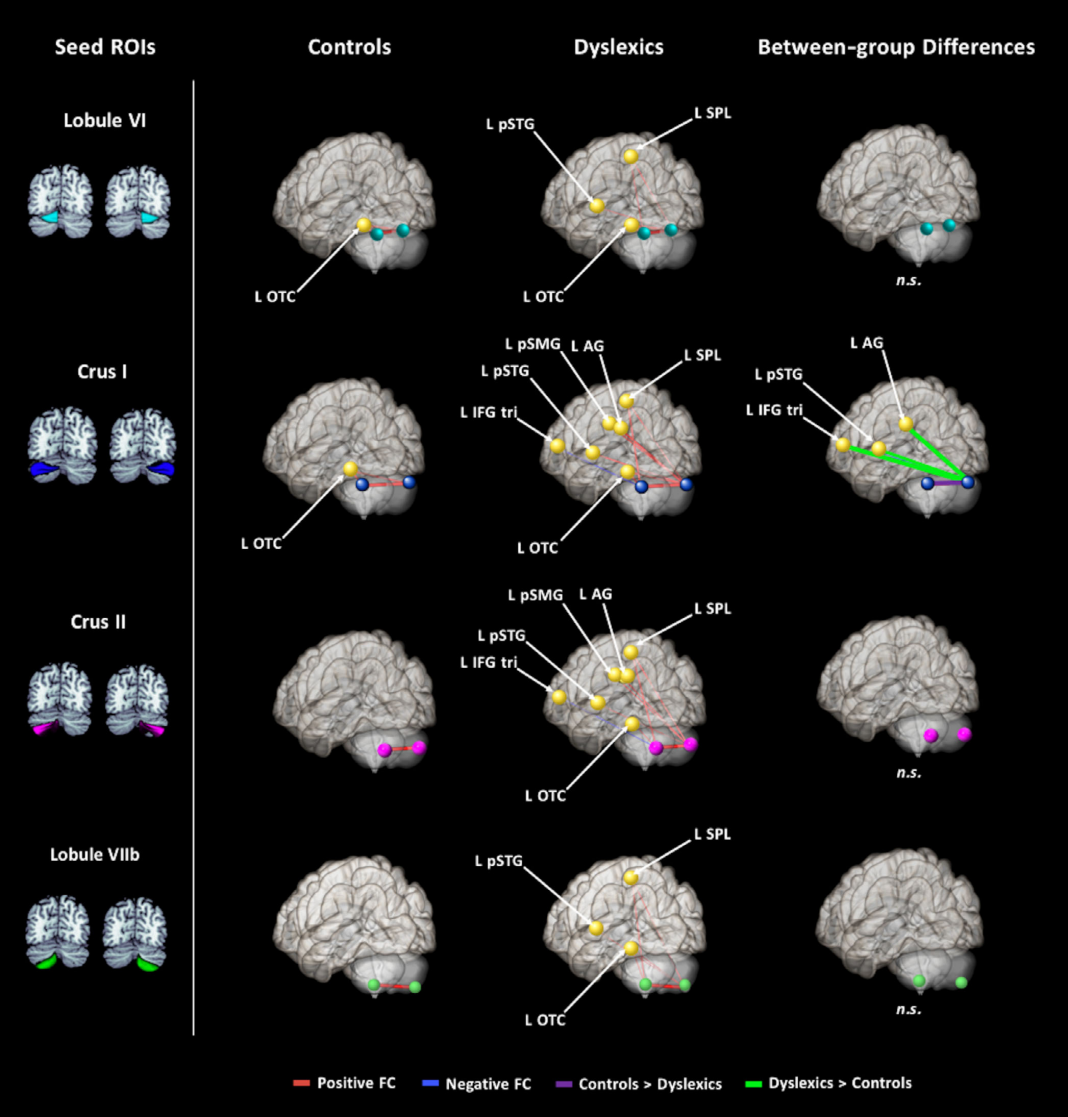
Background functional connectivity maps for left and right cerebellar seed regions: lobule VI, crus I, crus II, and lobule VIIb. Cortical target regions, depicted as spheres, are from the Harvard‐Oxford Atlas. Cerebellar seed regions were selected from the SUIT Atlas. In Controls, FC was largely limited to within the cerebellum, and between the cerebellum and left occipital‐temporal cortex. Dyslexics had FC from left and right cerebellar seed regions to several left hemisphere cortical regions, including posterior superior temporal gyrus and superior parietal lobule. Controls > dyslexics is shown in purple, while dyslexics > controls are shown in green. All results corrected for multiple comparisons, p‐FDR < .05, and two‐sided statistic. FC, functional connectivity

To test whether our predetermined cerebellar seed regions have FC with predetermined cortical target regions (Figure 1) independent of word processing, we examined background FC (Figure 4; Table 5). In controls, left lobule VI showed positive FC with right lobule VI and left occipital temporal cortex. Right lobule VI, showed positive FC with left lobule VI and left occipital temporal cortex. Left crus I had positive FC with right crus I and left occipital temporal cortex. Right crus I had positive FC with left crus I and left occipital temporal cortex. Left crus II had positive FC with right crus II; likewise, right crus II showed positive FC with left crus II. Left lobule VIIb had positive FC with right lobule VIIb; likewise, right lobule VIIb showed positive FC with left lobule VIIb.

**Table 5:** Background functional connectivity results

	Controls		Dyslexics		Between‐group differences	
Seed region	FC with…	T(22)	FC with…	T(22)	FC with…	T(43)
Left lobule VI	R lobule VI	14.00	R lobule VI	15.32		
	L OTC	10.66	L OTC	12.11		
			L SPL	3.26		
Right lobule VI	L lobule VI	14.00	L lobule VI	15.32		
	L OTC	8.99	L OTC	10.74		
			L SPL	4.39		
			L pSTG	2.86		
Left crus I	R crus I	11.01	R crus I	9.80	R crus I	2.96
	L OTC	4.07	L OTC	3.88		
			L SPL	4.24		
			L IFG tri	−2.78		
Right crus I	L crus I	11.01	L crus I	9.80	L crus I	2.96
	L OTC	4.18	L OTC	3.83		
			L SPL	3.82		
			L AG	5.83	L AG	−2.76
			L pSMG	3.37		
			L pSTG	3.87	L pSTG	−2.89
					L IFG tri	−2.83
Left crus II	R crus II	12.92	R crus II	13.32		
			L OTC	2.47		
			L SPL	4.74		
			L IFG tri	−3.02		
Right crus II	L crus II	12.92	L crus II	13.32		
			L SPL	4.98		
			L AG	3.35		
			L pSMG	3.32		
			L pSTG	2.34		
Left lobule VIIb	R lobule VIIb	14.20	R lobule VIIb	16.84		
			L OTC	3.33		
			L SPL	4.82		
Right lobule VIIb	L lobule VIIb	14.20	L lobule VIIb	16.84		
			L OTC	2.64		
			L SPL	4.46		
			L pSTG	2.60		

*Note*: Significance determined by seed‐level correction, *p*‐FDR < .05. Positive/Negative *t*‐statistics represent positive/negative connectivity. Positive *t*‐statistics in the between‐group differences column indicate controls > dyslexics, and negative *t*‐statistics indicate dyslexics > control. See Figure 5 for more details on between‐group differences.

Abbreviations: AG, angular gyrus; FC, functional connectivity; IFG, inferior frontal gyrus; L, left; OTC, occipital‐temporal cortex; R, right; pSMG, posterior supramarginal gyrus; pSTG, posterior superior temporal gyrus; RAN, rapid automatized naming; SPL, superior parietal lobule.

#### 3.3.2 Dyslexics

In the group with dyslexia, left lobule VI had positive FC with right lobule VI, left occipital temporal cortex, and left superior parietal lobule. Right lobule VI had positive FC with left lobule VI, occipital temporal cortex, superior parietal lobule, and posterior superior temporal gyrus (Figure 4; Table 5). Left crus I revealed positive FC with right crus I, occipital temporal cortex and left superior parietal lobule, and negative FC with left IFG *pars triangularis*. Right crus I had positive FC with left crus I, occipital temporal cortex, superior parietal lobule, angular gyrus, posterior supramarginal gyrus, and posterior superior temporal gyrus. Left crus II had positive FC with right crus II, left occipital temporal cortex, left superior parietal lobule, as well as negative FC with left inferior frontal gyrus *pars triangularis*. Right crus II had positive FC with left crus II, superior parietal lobule, angular gyrus, posterior supramarginal gyrus, and posterior superior temporal gyrus. Left lobule VIIb had positive FC with right lobule VIIb, left occipital temporal cortex, and left superior parietal lobule. Lastly, right lobule VIIb had positive FC with left lobule VIIb, occipital‐temporal cortex, superior parietal lobule, and posterior superior temporal gyrus.

#### 3.3.3 Controls versus dyslexics

**Figure 5:**
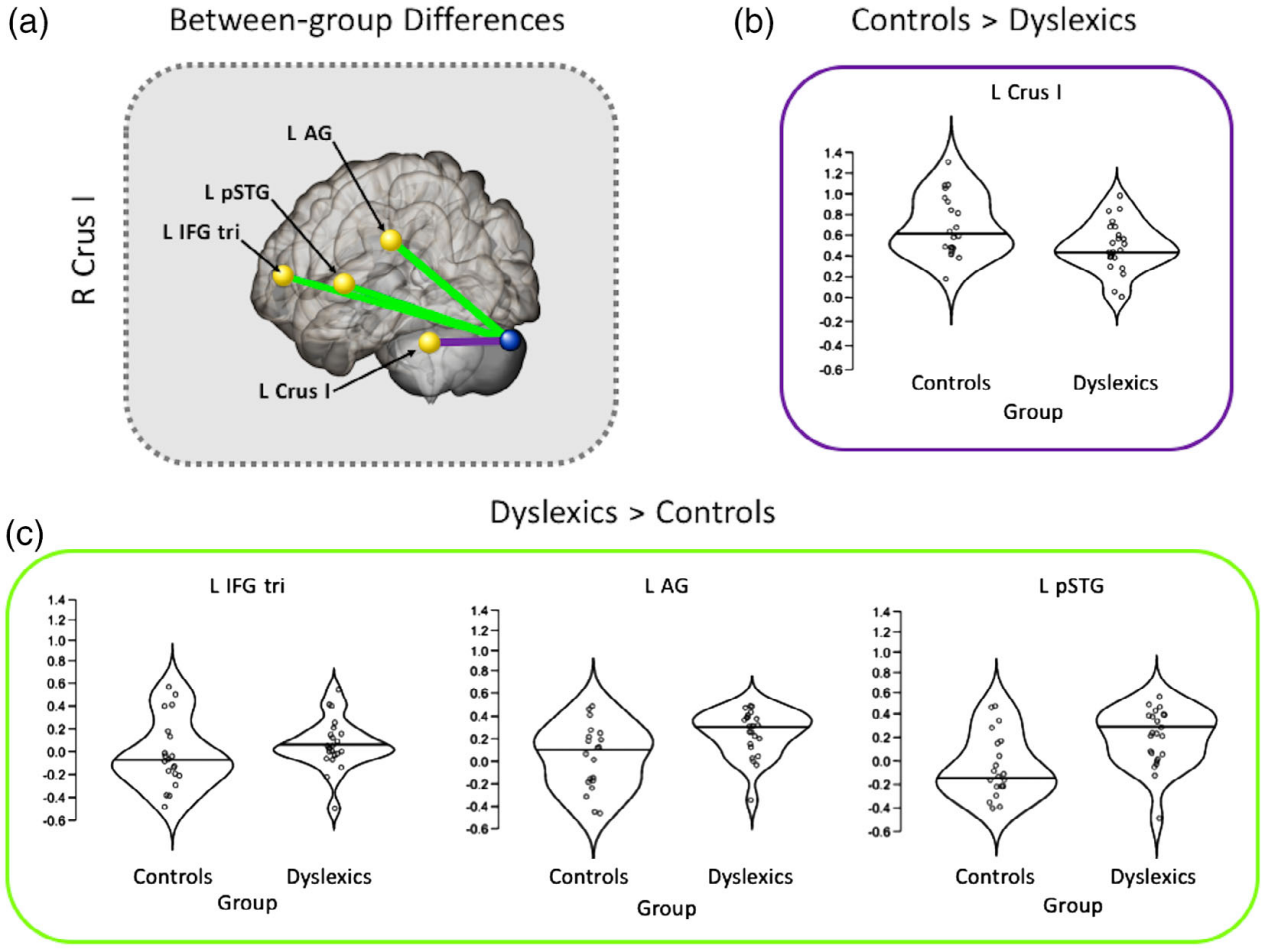
Background functional connectivity between‐group differences for right crus I examined in more detail. Taken from Figure 4, (a) Between‐group differences, where purple indicates positive FC Controls > Dyslexics and green indicates positive FC dyslexics > controls. Violin plots of *z*‐scores (y‐axis) for right crus I with (b) left crus I and (c) the three left cortical regions, IFG *tri*, AG, and pSTG. Black bars represent the mean of *z*‐score values and circles are *z*‐scores of individual participants. FC, functional connectivity; IFG, inferior frontal gyrus; pSTG, posterior superior temporal gyrus

Controls had greater positive FC between left crus I and right crus I (and vice versa). On the other hand, children with dyslexia had greater positive FC than controls between right crus I and left angular gyrus, posterior superior temporal gyrus, and inferior frontal gyrus *pars triangularis* (Figure 4; Table 5). Note that of these the left angular gyrus and posterior superior temporal gyrus (but not inferior frontal gyrus) was found in the group with dyslexia in the within‐group analysis. Figure 5 displays the distribution of the extracted *z*‐scores for each of these findings.

### 3.4 Task‐specific functional connectivity (gPPI) analysis of cerebellar seed regions

#### 3.4.1 Controls

The gPPI analysis, conducted to determine FC between cerebellar seed and cortical target regions (same regions as described above) specific to word processing, revealed no significant results in the controls.

#### 3.4.2 Dyslexics

There were no significant results.

#### 3.4.3 Controls versus dyslexics

There were no significant results.

### 3.5 Overall summary of results

While there was activity in the control group for RW > Fixation contrast (both at the level of the whole cerebellum and for the cerebellar sub‐regions), there was no significant activation in the control group specific to reading (RW > FF). The group with dyslexia revealed no activation for any of these analyses. Importantly, there were no between‐group differences. Turning to FC, there was background FC in both groups and some of these emerged in the between‐group comparisons, most notably right crus I and its greater positive functional connections to two left temporal–parietal regions in the group with dyslexia compared to controls. However, there was no task‐specific (gPPI) cerebellar FC with cortical regions in either group, and no between‐group differences.

## 4 DISCUSSION

This study tested the cerebellum's involvement in single word processing in children with and without developmental dyslexia. Based on the cerebellar deficit hypothesis (R. I. Nicolson et al., 2001), one would expect cerebellar activation in typically reading children during word processing and relative differences in children with dyslexia. Our results revealed no cerebellar activity in the typical readers or in the group with dyslexia specific to word processing, and there were no differences between the two groups. The cerebellar deficit hypothesis would also predict functional connectivity between the cerebellum and cortical regions known to be involved in reading, particularly left inferior frontal gyrus. However, there was no functional connectivity specific to word processing in either group and no between‐group differences. Below we offer interpretations of these results in the context of the cerebellar deficit hypothesis and the literature on the neural bases of reading.

### 4.1 The cerebellar deficit hypothesis as an explanation for impaired reading

The cerebellar deficit hypothesis posits that cerebellar dysfunction causes poor acquisition of early articulatory skills and in turn impairs phonological processing, thereby causing the reading difficulties observed in dyslexia (R. I. Nicolson et al., 2001). This proposed role of the cerebellum seems plausible given the cerebellum's role in various linguistic processes (Ackermann, Mathiak, & Riecker, 2007; De Smet, Paquier, Verhoeven, & Mariën, 2013; Mariën et al., 2013), the functional connections between the right cerebellum and left inferior frontal cortex found in typical adults (James R. Booth, Wood, Lu, Houk, & Bitan, 2007), and the left inferior frontal gyrus' involvement in both articulation and phonology (Burton, 2001). Based on this, we expected to find cerebellar activation and functional connectivity to left IFG or other regions known to be involved in reading during word processing in typical children. Only a few studies have demonstrated differences in activation of the cerebellum during reading in dyslexia in adults (Brambati et al., 2006; Brunswick, McCrory, Price, Frith, & Frith, 1999; Richlan et al., 2010; Rumsey et al., 1997) and in children (Feng et al., 2017; Hu et al., 2010; Meyler, Keller, Cherkassky, Gabrieli, & Just, 2008; Temple et al., 2001). However, consistent with other publications and as discussed next in more detail, we found no differences in activity or functional connectivity between our two groups during word processing.

### 4.2 Activation in typical readers

In the control group, there was activation in vermis VI, left crus I, and right lobule VI when comparing RW processing with Fixation at the level of the whole cerebellar analysis. The same comparison at the level of the cerebellar sub‐regions, revealed similar results: left and right lobule VI, and left crus I, as one would expect given that the locations of these sub‐regions coincided with areas of activation in the whole cerebellum analysis. However, when contrasting RW with FF processing, we did not find activity for the analysis of the whole cerebellum or its sub‐regions, suggesting that the cerebellum is not specifically involved in reading.

While prior studies in typically reading children have not focused specifically on the cerebellum, there is a corpus of studies examining whole brain activity during reading and reading‐related tasks. A comprehensive meta‐analysis of reading‐related studies in typical readers of alphabetic languages showed no concurrence for cerebellar activation in typical children (Martin et al., 2015). This result and that of the current study fails to support a potential role of the cerebellum in normal reading in children. It is worth noting that only six (from five studies) of the 20 original experiments included in the meta‐analysis reported cerebellar activity during reading‐related tasks (J. R. Booth et al., 2001; Gaillard, Balsamo, Ibrahim, Sachs, & Xu, 2003; Hoeft et al., 2006; Noble, Wolmetz, Ochs, Farah, & McCandliss, 2006; Rimrodt et al., 2009). In addition to these studies included in this meta‐analysis, a study of Chinese children found cerebellar activation (bilateral crus I, right crus II) during a phonological task and cerebellar activation (bilateral crus I, right lobule VI) during an orthographic task, when contrasted with fixation (Feng et al., 2017). Interestingly, this study and all the 20 experiments included in the meta‐analysis by Martin et al. (2015) used a low‐level comparison condition, suggesting that any cerebellar activation during reading‐related task found in these studies could be due to motor‐related functions of the task (e.g., eye‐movements during reading or button pressing). Our findings are consistent with this, revealing activity of the cerebellum in typical readers when the single word processing task is contrasted with fixation, but not when contrasted with the active control task matched on motor‐related processing.

While not examined here, it is important to consider that our task does reveal cortical regions known to be involved during reading. A prior publication using the same tasks (and a subset of the participants studied here) revealed that typically reading children activate left cortical regions (left inferior frontal and fusiform gyri), indicating that cortical areas involved in word processing are identified by this contrast (O. A. Olulade et al., 2015). The same study also reported reduced activation within the fusiform gyrus in children with dyslexia, similar to other studies, as will be discussed further in the next section. Taken together, even though the current study focused its analyses on the cerebellum, our results do not support the notion that the cerebellum is involved in word processing.

### 4.3 Activation in children with dyslexia

The cerebellar deficit hypothesis would predict a difference in activation of the cerebellum during single word processing in children with dyslexia. Our results showed no activation for children with dyslexia when contrasting RW reading to Fixation, or when contrasting it to FF, for both the whole cerebellum and sub‐regions of interest analyses. Importantly, there were no between‐group differences. There are three meta‐analyses reports capturing many original studies that compare groups with and without dyslexia during reading‐related tasks (Linkersdörfer et al., 2012; Maisog et al., 2008; Richlan et al., 2011). As reviewed in detail next, none of these meta‐analyses reported less activity in the cerebellum in children with dyslexia either.

Maisog et al. (2008) found likelihood of less activation in adolescents and adults with dyslexia compared to controls in cortical regions including left inferior parietal cortex, bilateral superior temporal gyrus and left inferior frontal gyrus, as well as more activation in right thalamus and right anterior insula (mean ages 14 to 43 years). Six of the nine studies did not report differences in cerebellar activation in adults/adolescence with dyslexia (Georgiewa et al., 1999; Grunling et al., 2004; Ingvar, Eriksson, & Stone‐Elander, 2002; McCrory, 2004; E. Paulesu, 2001; Eraldo Paulesu et al., 1996). However, two reported under‐activation in dyslexia within the cerebellum, one during an orthographic decision task in contrast to eyes closed at rest in adults (Brunswick et al., 1999) and another during an aloud reading task (Flowers, Georgetown University, Washington DC, unpublished data). The third found more cerebellar activation during phonological decision‐making in contrast to fixation in adults with dyslexia (Rumsey et al., 1997).

The meta‐analysis by Richlan et al. (2011) on studies that compared children with and without dyslexia (mean ages 9.4 to 11.6 years) included eight published fMRI studies and one poster, the results of which were published later (none of which overlapped with Maisog et al., 2008). The study found no differences in cerebellar activation in dyslexia, but there was convergence of under‐activation in left inferior parietal lobule, supramarginal gyrus, and fusiform gyrus in children with dyslexia relative to controls. Of the eight studies that produced these results, six did not report altered cerebellar activation relative to controls (V. Blau et al., 2010; J. R. Booth, Bebko, et al., 2007; Cao, Bitan, Chou, Burman, & Booth, 2006; Hoeft et al., 2006; Schulz et al., 2009; van der Mark et al., 2009). Only two found differences in cerebellar activation; specifically these studies reported *more* cerebellar activation in dyslexia in comparison to controls (Meyler et al., 2008; Temple et al., 2001). These studies involved letter matching in contrast to line matching (Temple et al., 2001) and sentence reading in contrast to fixation (Meyler et al., 2008).

Note that the same report by Richlan et al. (2011) also included a meta‐analysis on studies of adults with and without dyslexia (nine studies) and found no convergence of under‐ or over‐activations in the cerebellum. Four of these studies were included in the Maisog et al. (2008) meta‐analysis (Ingvar et al., 2002; E. Paulesu, 2001; Eraldo Paulesu et al., 1996; Rumsey et al., 1997). Of those not previously discussed, three found no differences between the groups in cerebellar activation (Blau, van Atteveldt, Ekkebus, Goebel, & Blomert, 2009; McCrory, 2004; Wimmer et al., 2010). Of the two remaining studies, one found more cerebellar activation during pseudoword reading in comparison to fixation (Richlan et al., 2010) and the other found less cerebellar activation during silent reading in comparison to viewing false font strings (Brambati et al., 2006). This work was a follow‐up study from Richlan et al., 2009, for which meta‐analyses of children and adults had been combined and did not reveal either under‐ or over‐activation in dyslexia in the cerebellum.

Finally, Linkersdörfer et al. (2012) conducted a series of meta‐analyses in children and adults with and without dyslexia. The overall goal was to co‐localize findings from functional studies with those from anatomical (gray matter volume) studies. Their first meta‐analysis of functional studies combined children and adults with dyslexia (24 studies in total; mean ages of 5.9 to 31.6 years), and revealed more activation in left cerebellum (lobule VI) during reading‐related tasks in dyslexia among other between‐group differences (Linkersdörfer et al., 2012). Two of these studies were included in Maisog et al. (2008) meta‐analysis (Georgiewa et al., 1999; Grunling et al., 2004), eight in the meta‐analysis by Richlan et al. (2011) for children, and nine in the meta‐analysis by Richlan et al. (2011) for adults. Of the five studies not previously discussed, four studies, three in children (Hoeft et al., 2007; Kronbichler et al., 2006; Maurer et al., 2011), and one in adults (Pecini et al., 2011), did not report differences between those with and without dyslexia in the cerebellum. The last study reported less activation during semantic word matching relative to fixation in children with dyslexia in left lobule I‐IV (Hu et al., 2010). Next, meta‐analyses were conducted for studies of children and adults separately, and these were limited to those studies reporting under‐activation in dyslexia during reading‐related tasks. Both of these meta‐analyses comparing groups with and without dyslexia in either children (9 studies) or adults (10 studies), all of which were previously included in either the meta‐analysis by Maisog et al. (2008) or Richlan et al. (2011), revealed no differences in activity the cerebellum.

In sum, of the 12 studies in children reviewed above, only one study has shown less cerebellar activity in children with dyslexia compared to controls in alphabetic languages (Hu et al., 2010) and two have shown more activity in groups with dyslexia compared to controls (Meyler et al., 2008; Temple et al., 2001). While the current study focuses on children, it is interesting to consider the findings in adults: of the 15 studies reviewed above, only two published studies (Brambati et al., 2006; Brunswick et al., 1999) and one unpublished report (Flowers, Georgetown University, Washington DC, unpublished data) have shown less cerebellar activity in dyslexia compared to controls in alphabetic languages; two showed more activity in adults with dyslexia (Richlan et al., 2010; Rumsey et al., 1997). Thus, the results from prior studies and the current study, which employed analyses focused specifically on the cerebellum, fail to show convincing evidence that suggests less cerebellar activation in children. The only study that did find less activation in the cerebellum in children, used a use low‐level fixation comparison task and did not correct the statistical map for multiple comparisons (Hu et al., 2010). Taken together, the evidence from brain imaging studies seem to converge in their failure to support the cerebellar deficit hypothesis.

### 4.4 Functional connectivity

Based on the cerebellar deficit hypothesis, we would expect to find cerebellar connectivity during reading between the cerebellum and the left IFG in typically developing children, and alteration of these in children with dyslexia. We first examined background connectivity to assess whether there were any aberrations in intrinsic FC not specific to reading. Then we used a gPPI FC analysis to test for FC modulated specifically during single word reading. We expected FC specific to the word processing task in the typical readers between the cerebellum and frontal regions as affirmation of a cerebellar‐frontal loop underlying successful reading, and altered FC in the group with dyslexia. We found background FC within and between groups. However, we found no task‐specific cerebellar FC with IFG or any other cortical target regions during word processing in either group, and no between‐group differences.

### 4.5 Intrinsic connectivity in typical readers

In the present study, we found that left and right crus I and lobule VI had positive background (intrinsic) FC with left occipital‐temporal cortex in the typically reading children. It is important to note that background connectivity (Norman‐Haignere et al., 2012), sometimes referred to as pseudo‐resting state (Sheffield et al., 2015), has similarities with resting‐state connectivity (Fair et al., 2007) and as such prior findings using resting‐state connectivity focused on the reading network are of relevance to the discussion of the results of the present study. Most of these previous studies have not reported resting‐state connectivity between the cerebellum and cortex (Hampson et al., 2006; Koyama et al., 2010; Zhao et al., 2011); however, this can largely be attributed to the common use of cortical, and not cerebellar, seed regions. Nevertheless, the literature offers some evidence of cerebellar FC with cortical regions that contribute to reading. For example, a recent publication using Neurosynth (an online functional connectivity tool akin to a meta‐analysis) found that cerebellar lobule VIIb/Crus II has resting state FC with intraparietal lobule (Alvarez & Fiez, 2018). Furthermore, a resting‐state connectivity study used a data‐driven approach to divide cortex into seven general networks and then determined how these networks map onto the cerebellum (Buckner et al., 2011). These researchers found that two of these cortical networks, frontal–parietal and ventral attention networks (including cortical reading‐related regions such as inferior frontal gyrus, temporal–parietal cortex, and occipital‐temporal cortex), mapped onto regions of the cerebellum including lobule VI and crus I (Buckner et al., 2011). Thus, our FC finding between cerebellum and occipital‐temporal cortex is consistent with this specific resting‐state study.

### 4.6 Intrinsic connectivity in children with dyslexia

Within‐group maps for children with dyslexia revealed cerebellar background FC with multiple regions of the reading network. Of note, right crus I had positive FC with left occipital temporal cortex, superior parietal lobule, angular gyrus, posterior supramarginal gyrus, and posterior superior temporal gyrus. Left crus I had positive FC with left occipital temporal cortex and superior parietal lobule, and negative FC with left inferior frontal gyrus *pars triangularis*.

When comparing cerebellar background functional connectivity between the two groups, those with dyslexia had greater positive FC between right crus I and left angular gyrus, posterior superior temporal gyrus, and left inferior frontal gyrus *pars triangularis*. Of these, the connections from the cerebellum to the left angular gyrus and posterior superior temporal gyrus were consistent with the positive FC found in the dyslexic within‐group results. Although the FC between right crus I and left inferior frontal gyrus *pars triangularis* was stronger in the group with dyslexia than the controls in the between‐group comparison, there was no FC between these regions in the within‐group maps for either groups. The mean FC *z*‐score values show that the higher FC in the dyslexic group in the left inferior frontal gyrus *pars triangularis* was a product of the negative FC in the controls and positive FC in the children with dyslexia (see Figure 5). Given that the cerebellar deficit hypothesis emphasizes a developmental impairment in children with dyslexia, one would have predicted weaker background FC in the group with dyslexia rather than stronger, but there were no findings of relatively less FC in dyslexia.

When considering the findings of stronger background FC between right crus I and left angular gyrus, and posterior superior temporal gyrus in dyslexia, it should be noted that the resting‐state connectivity literature reports only relatively *less* functional connectivity in groups with dyslexia. One study found less FC between bilateral cerebellum (lobule VI/crus I and crus II) and left parietal lobule in young adults with dyslexia in comparison to controls (Schurz et al., 2015). Other studies, where the cerebellum was not included in the analyses, found that children with dyslexia had less connectivity between cortical regions, specifically left intraparietal sulcus and left middle frontal gyrus (Koyama et al., 2013), left occipital‐temporal and left inferior parietal regions (Finn et al., 2014; van der Mark et al., 2011), and left occipital‐temporal and left occipital regions (Finn et al., 2014). Again, while these studies may not have had findings in the cerebellum due to the choice of a cortical seed regions, the present study reveals, even when testing seeds within the cerebellum specifically, that intrinsic FC between the cerebellum and IFG is not weaker in dyslexia compared to controls as would be predicted by the cerebellar deficit hypothesis.

One possibility for these observations of greater FC between right crus I and left angular gyrus, and posterior superior temporal gyrus in dyslexia is that this hyper‐connectivity at baseline in dyslexia is driven by a network that shares parts of, or all of these connections. A study by Bernard et al. (2012) speaks to the idea that cerebellar lobules have resting state connectivity with multiple cortical regions in typical adults. Of relevance to our study, Bernard et al., found that resting‐state networks from right cerebellar seed regions, including a positive functional connection between right crus I and left angular gyrus as well as right inferior parietal lobule. While we did not observe such FC in our typically reading group, it nevertheless confirms the notion that these regions are functionally connected at rest, at least in typical adults, and that these may be altered in dyslexia. Alternatively, the observed differences in baseline FC could reflect the influence of other cortical regions that are adversely impacted by dyslexia. For example, the posterior superior temporal gyrus differs in brain anatomy and function in dyslexia. Anomalies here are likely to be perpetuated through functional connections to other regions outside of the network for reading, which may include the cerebellum, thereby leading to baseline differences in FC. In both scenarios, the cerebellum is still not likely not be the “culprit,” but rather an “innocent bystander” (Zeffiro & Eden, 2001).

Our prediction for weaker functional connectivity in the group with dyslexia was based on the premise of the cerebellar deficit hypothesis. However, more broadly speaking developmental impairments have been attributed to the lack of pruning (Berl, Vaidya, & Gaillard, 2006), which may lead to abnormal functional connectivity patterns manifested as either hyper‐ or hypo‐connectivity in comparison to typical readers. The background hyper‐connectivity observed in the group with dyslexia could be explained in these terms, although causality would still not be attributed to the cerebellum per se. Another consideration is that neurodevelopmental alteration in FC strength and pattern of network interactions are thought to change from childhood to adulthood (Ernst, Torrisi, Balderston, Grillon, & Hale, 2015), suggesting that future investigations will benefit from studies like ours at different age groups.

### 4.7 Functional connectivity specific to reading in children with and without dyslexia

As discussed for background FC, when it comes to examining correlations between brain regions during reading, prior studies have emphasized a *deficit* in the connections between brain regions known to be involved in reading. For example, Paulesu et al. (1996) used their findings of reduced activation within left inferior frontal gyrus and supramarginal gyrus during a phonological short‐term memory task to propose weaker connectivity in dyslexia, noting a “disconnection” (although they did not actually measure correlations between the two regions). A more recent study found hypo‐connectivity between left inferior frontal gyrus and left superior temporal gyrus (Boets et al., 2013), and revisited the notion of disconnections among cortical brain regions, even for those that were showing normal activation in the group with dyslexia.

While the cerebellar deficit hypothesis would predict altered FC between the cerebellum and left IFG during word processing, this prediction was not confirmed. Altogether, we found no within‐ (control or children with dyslexia) nor between‐group connectivity that was modulated during single word processing (gPPI analysis). Few prior studies have examined task‐specific FC during a reading task and in dyslexia. An early PET study of adults with dyslexia reported lower correlations of left angular gyrus with left inferior frontal superior temporal, and fusiform gyri as well as left cerebellum during pseudoword reading. This study also found lower correlations between left angular gyrus and left superior temporal and fusiform gyri, and left cerebellum during exception word reading (Horwitz, Rumsey, & Donohue, 1998). Another relevant study discussed above in terms of resting‐state FC, also performed a task‐based FC analysis and found that young adults with dyslexia had reduced FC in comparison to controls between left parietal lobule and right cerebellum (lobule VI/crus I and crus II) during silent reading and phonological lexical decision tasks (Schurz et al., 2015). While these studies were conducted in adults with dyslexia, there are few equivalent studies in children. One of the few studies used seed‐to‐voxel connectivity analysis during a phoneme‐mapping task and found no significant differences in their cerebellar seed region of interest with cortical regions but did find differences with the seed in left inferior frontal gyrus (Richards & Berninger, 2008). A study in Chinese children showed that FC strength between left cerebellum and left supramarginal gyrus was stronger in children with dyslexia (than in controls) during a phonological task, but at the same time FC between right cerebellum and left fusiform gyrus was weaker during an orthographic task (Feng et al., 2017).

Taken together, we found intrinsic (background) hyper‐connectivity of right crus I with two cortical reading‐related regions in the group with dyslexia relative to controls (left angular gyrus and posterior superior temporal gyrus), but no such hyper‐connectivity during single word processing. As such, the results indicate the difference is not specific to single word processing.

### 4.8 Lack of support for the cerebellar deficit hypothesis: Interpretation of the null result

While our results do not support the cerebellar deficit hypothesis of dyslexia, it behooves us to consider alternative interpretations of null result. The present study employed an implicit reading task that is designed to activate areas involved in orthographic, semantic, and phonological processing. However, it is worth considering whether an implicit reading task requires similar demands for phonological processing as an explicit reading task. Prior publications from our lab that use this implicit reading task in typically reading children have reported activation in areas known to be involved in phonological processing, including left superior temporal cortex (Turkeltaub et al., 2003); left inferior frontal gyrus (Olulade et al., 2015; Olulade et al., 2013); and left superior temporal and inferior frontal cortices (Evans et al., 2016). When using this task to compare children with and without dyslexia, we found differences in left fusiform gyrus, left insula, right lingual gyrus, and right superior temporal gyrus (O. A. Olulade et al., 2015). There is other support for this kind of task eliciting differences between those with and without dyslexia. A study by Brunswick et al. (1999) found less activation in adults with dyslexia in comparison to adult controls in left inferior/middle temporal, left frontal operculum/anterior insula, and bilateral cerebellum (a sub‐region that is most likely lobule VI) when combining explicit and implicit word reading tasks. When looking at each task, less activation was found in the dyslexic group during explicit word reading (reading aloud) in left cerebellum, medial extrastriate/lingual gyrus, basal temporal, caudate/thalamus, and premotor area; and during implicit word reading (similar to our task) in left posterior basal temporal region boarding the cerebellum, left inferior temporal, left middle temporal, and left inferior parietal areas. Brunswick et al., used these results to argue that the within‐group activation patterns, as well as between‐group differences in activation, are similar regardless of whether an explicit or implicit reading task is used, which should mitigate any concern that differences between implicit and explicit tasks drives the null results found in our study. Nevertheless, future studies will need to look at adults and children with dyslexia using the implicit and explicit reading task to confirm or refute this possibility.

Our lack of evidence for the cerebellar deficit hypothesis is consistent with the limited support for this theory. As recently pointed out by Nicolson and Fawcett (2019), the framework has drawbacks, such as the problems of truly isolating cerebellar function from cerebral functions. Also, longitudinal studies are needed and the role of comorbid disorders need to be elucidated. The authors of the cerebellar deficit hypothesis have revised their framework to a “delayed neural commitment framework,” implicating the cerebellum in slower skill acquisition and slower building of neural networks or alternative networks that subserve reading (R. I. Nicolson & Fawcett, 2019). As such, the framework has broadened in its scope, including learning networks involving sensori‐motor‐cognitive integration, thereby proposing dysfunction of the very networks investigated in the present study. However, as noted above, there is only one study in children using alphabetic languages to date that shows less activation in the cerebellum (Hu et al., 2010); and in adults there are only two (Brambati et al., 2006; Brunswick et al., 1999). Taken together our results are consistent with the published literature and do not support the cerebellum as a brain structure whose impairment is responsible for developmental dyslexia.

## 5 CONCLUSIONS

We tested the cerebellar deficit hypothesis of dyslexia by measuring activity and functional connectivity during single word processing in typically reading children and those with dyslexia. In a region of interest analysis examining the cerebellum, and also sub‐regions of the cerebellum, we found no activity specific to reading in either group, and no between‐group differences. Moreover, we did not find FC specific to single word processing in controls or dyslexic children and no between‐group differences. Overall, our results do not support the cerebellar deficit hypothesis of dyslexia.
